# Emerging Dementia Biofluid Biomarker Candidates Identified in Human Proteomic Studies (2021‐2025)

**DOI:** 10.1002/alz70856_106999

**Published:** 2026-01-08

**Authors:** Anne‐Li TB Lind

**Affiliations:** ^1^ Olink Proteomics, Uppsala, Uppsala, Sweden

## Abstract

**Background:**

Recent proteomic analyses of biofluids have identified numerous emerging dementia biomarker candidates.

**Method:**

We reviewed 39 dementia proximity extension assay (PEA) studies of cerebrospinal fluid (CSF) and blood (including UK Biobank data) published between 2021 and 2025 to identify trends in emerging dementia biomarkers.

**Results:**

Our analysis revealed that the 39 dementia studies identified over 180 proteins regulated in human biofluids. Multiple studies confirmed the value of established biomarkers such as GFAP (astrocytic activation), NFL (neurodegeneration), and IL6 (inflammation). Twenty‐six proteins, including IL6, MMP10, GDF15, NEFL, CDCP1, ITGB2, CXCL9, IGFBP2, and GFAP, were identified in multiple studies, while 104 proteins were uniquely identified in a single study. Thirteen publications utilized UK Biobank data. We highlighted key pathways and trends in emerging dementia biofluid biomarkers based on PEA analysis. These studies focused on various biomarker types spanning at least four FDA biomarker classes: risk, diagnostic, prognostic, and pharmacodynamic.

**Conclusion:**

Recent biofluid proteomic studies have identified novel risk, diagnostic, prognostic, and pharmacodynamic biomarker candidates for cognitive decline, warranting further validation.

